# *Plasmodium* parasitophorous vacuole membrane protein Pfs16 promotes malaria transmission by silencing mosquito immunity

**DOI:** 10.1016/j.jbc.2023.104824

**Published:** 2023-05-15

**Authors:** Julian Ramelow, Yacob Keleta, Guodong Niu, Xiaohong Wang, Jun Li

**Affiliations:** 1 Biomedical Sciences Graduate Program, Herbert Wertheim College of Medicine, Florida International University, Miami, Florida, USA; 2 Department of Biological Sciences, Florida International University, Miami, Florida, USA; 3 Biomolecular Sciences Institute, Florida International University, Miami, Florida, USA

**Keywords:** Pfs16, mosquito, malaria, vector-borne diseases, innate immunity, JNK pathway, immune modulation

## Abstract

With rising cases for the first time in years, malaria remains a significant public health burden. The sexual stage of the malaria parasite infects mosquitoes to transmit malaria from host to host. Hence, an infected mosquito plays an essential role in malaria transmission. *Plasmodium falciparum* is the most dominant and dangerous malaria pathogen. Previous studies identified a sexual stage-specific protein 16 (Pfs16) localized to the parasitophorous vacuole membrane. Here, we elucidate the function of Pfs16 during malaria transmission. Our structural analysis identified Pfs16 as an alpha-helical integral membrane protein with one transmembrane domain connecting to two regions across parasitophorous vacuole membrane. ELISA assays showed that insect cell-expressed recombinant Pfs16 (rPfs16) interacted with *Anopheles gambiae* midguts, and microscopy found that rPfs16 was bound to midgut epithelial cells. Transmission-blocking assays demonstrated that polyclonal antibodies against Pfs16 significantly reduced the number of oocysts in mosquito midguts. However, on the contrary, feeding rPfs16 increased the number of oocysts. Further analysis revealed that Pfs16 reduced the activity of mosquito midgut caspase 3/7, a key enzyme in the mosquito Jun-N-terminal kinase immune pathway. We conclude that Pfs16 facilitates parasites to invade mosquito midguts by actively silencing the mosquito’s innate immunity through its interaction with the midgut epithelial cells. Therefore, Pfs16 is a potential target to control malaria transmission.

Malaria remains a significant global disease. In 2021, malaria case rates increased by 6% compared to the 2020 report (https://www.who.int/teams/global-malaria-programme/reports/world-malaria-report-2021), and Africa remains the epicenter of malaria occurrence, reporting 94% of cases and deaths. About 241 million cases and 627,000 deaths were officially reported; the undocumented numbers are presumably higher. Therefore, a better understanding of the pathogenesis of this devastating disease is still needed.

Several *Plasmodium* species, including *Plasmodium falciparum*, *Plasmodium vivax*, *Plasmodium malaria*, *Plasmodium ovale*, *and Plasmodium knowlesi*, are capable of infecting humans and causing the disease. *P. falciparum* accounts for approximately 99.7% of malaria cases in Africa and is the deadliest parasite (1, 2). When a malaria parasite invades an erythrocyte, a portion of the host cell membrane will be invaginated to form a parasitophorous vacuole membrane (PVM) around the parasite. The PVM is modified by the parasite and integrated by parasite proteins through secretory organelles during parasite development (3).

During the parasite development at the intraerythrocytic stage, some trophozoites experience sexual differentiation and develop into male and female gametocytes, which can infect anopheline mosquitoes, definitive hosts for parasite sexual reproduction (4, 5, 6). After a mosquito takes infected blood, gametocyte egress comprises a set of sequential events including rapturing the PVM and releasing gametes and PVM pieces (7). Female and male gametes form ookinetes to infect mosquitoes. Many factors inside mosquito midguts affect parasite transmission (8). In particular, several interactive mosquito or parasite proteins have been identified and linked to be crucial for parasite transmission to mosquitoes in either anchoring parasites (9, 10) or inducing immune evasions such as most recent Pfs47 and Pfs47Rec interaction (11, 12). One of the several emerging molecules is the PF3D7_0406200, commonly referred to as the sexual stage-specific protein 16 (Pfs16) (9). The expression of *Pfs16* is induced immediately following the invasion of a red blood cell in sexually committed ring-stage parasites and continues throughout gametocytogenesis (13). Notably, it is located primarily on the PVM of *P. falciparum* gametocytes (14). The Pfs16 gene knockout parasites showed a 4- to 5-fold reduction in gametocytes generated (15). *Pfs16* KO parasites failed to infect mosquitoes when compared to WT parasites (15).

Anopheles mosquitoes can deploy several effective antiplasmodial responses by activating innate immune signaling cascades (16, 17, 18). The Jun-N-terminal kinase (JNK) pathway (19), mediated by mitogen-activated protein kinases (20), involves various biological processes such as apoptosis, immunity, and cell proliferation by activating specific caspases (21, 22) and generates some effectors to inhibit parasite infection in mosquito midguts (23). Indeed, the caspase activities are used to monitor the activation of JNK pathways (24). The JNK pathway also interplays with other immune pathways including the immune deficiency and *Toll* pathways by sharing key proteins, such as transforming growth factor-β-activated kinase 1 (25, 26). Immune deficiency and *Toll* pathways mediate ookinete lysis (27).

Previous studies showed antibodies against Pfs16-inhibited *P. falciparum* transmission to mosquitoes (9). However, its specific role remains unknown. Several potential mechanisms exist where Pfs16 can exhibit its effects in malaria transmission to mosquitoes: (1) direct interaction to position parasites for midgut invasion, (2) enzymatic digestion to disrupt midgut physical barriers, (3) direct or indirect inhibition of the mosquito's innate immunity to promote transmission, among others. In this work, we found that Pfs16 facilitated malaria transmission by negatively regulating the JNK response in the mosquito immune activation pathway, increasing parasite oocysts in mosquito midguts. The findings provide insightful mechanisms of newly discovered parasite-mosquito interplay during malaria transmission and present Pfs16 as a prime candidate for transmission-blocking development.

## Results

### Pfs16 is an integrated membrane protein at PVM

**Figure 1:**
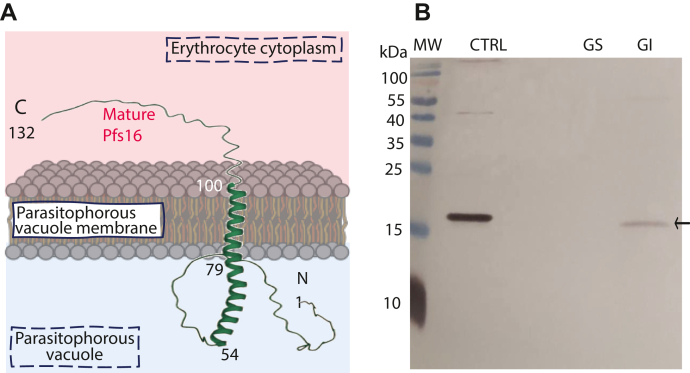
**Pfs16 structure prediction and cellular location.***A*, predicted Pfs16 structure and location. *B*, endogenous Pfs16 is confirmed in the gametocyte membrane fraction. Lanes: M, protein ladder; C, the pure rPfs16 protein; GS, gametocyte soluble fraction; GI, gametocyte insoluble fraction. rPfs16, recombinant Pfs16.

Pfs16 is 157 amino acid residues in length. SignalP (28) and TMHMM (29) predicted that Pfs16 consists of a signal peptide from residue 1 to 25 for protein-membrane trafficking and a transmembrane domain from residue 105 to 125 (89–100 in the mature protein form) for PVM integration. AlphaFold2 (30) predicted the structure of the mature Pfs16 that contains one long α-helix from residue 54 to 100. The Pfs16 protein is synthesized by the parasite and integrated into PVM with the help of translocon machinery (31). Taking together, the mature Pfs16 is an integrated membrane protein with the N-terminal inside parasitophorous vacuole and the C-terminal in erythrocyte cytoplasm, respectively (Fig. 1*A*).

To investigate the functional role of the Pfs16 protein, we previously generated rabbit polyclonal antibodies against *Escherichia coli*–expressed Pfs16 (9). Using the antibodies, we determined the endogenous Pfs16 in cellular fraction using this polyclonal antibody by Western blotting assays. In brief, *P. falciparum* gametocytes were lysed with Tris buffer to obtain the soluble fraction. After centrifugation, the supernatant contained soluble protein, and the insoluble pellet was extracted with Tris–Triton to extract the membrane proteins. The soluble and membrane proteins were separated by SDS-PAGE and analyzed by Western blotting. The result showed that endogenous Pfs16 protein was detected only in the membrane fraction of the parasite lysate and not in the soluble fraction (Fig. 1*B*), confirming that Pfs16 is a membrane protein.

### Expressing recombinant Pfs16 in insect cells

**Figure 2:**
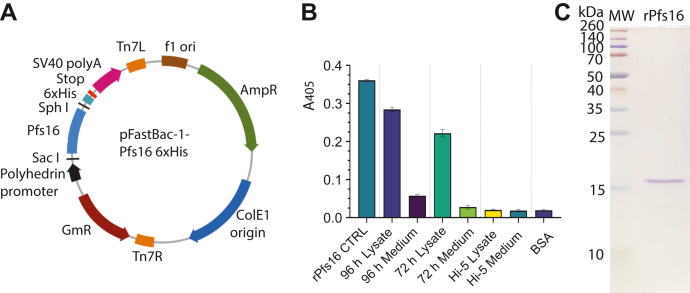
**Expressing and measuring rPfs16 protein in Hi5 cells.***A*, construct of the pFastBac1-Pfs16 vector used for baculovirus insect cell expression. *B*, expression of rPfs16 protein in medium and Hi5 cells; after 72 h and 96 h, expression was quantified with anti-His mAb by ELISA at 405 nm absorbance (*A*_405_). BSA, Hi5 cells, and precultured medium serve as control samples. The rPfs16 is the purified rPfs16 protein. *C*, the rPfs16 expressed in Hi5 cells was purified, showing only one band on the SDS-PAGE. Molecular weight (MW) depicts the protein markers. BSA, bovine serum albumin; Hi5, High Five; rPfs16, recombinant Pfs16.

We expressed recombinant Pfs16 protein (rPfs16) using the baculovirus expression system in High Five (Hi5) cells. The *Pfs16* was cloned into pFastBac (Fig. 2*A*), which generated a recombinant baculovirus that expressed rPfs16 in Hi5 cells. The cells were harvested at different times by centrifugation. Proteins in cells were extracted by native cell lysis buffer that contains detergent and protease inhibitors. The amount of rPfs16 in the culture medium and cells was quantified with anti-His mAb by ELISA. The results showed that rPfs16 is highly expressed in Hi5 cells at 72 h and 96 h (Fig. 2*B*) but not in the culture medium, which is consistent with endogenous Pfs16. The rPfs16 protein was isolated from the cells *via* FPLC chromatography using HisTrap affinity column to a single major band on SDS-PAGE (Fig. 2*C*).

### The effects of Pfs16 on malaria transmission

**Figure 3:**
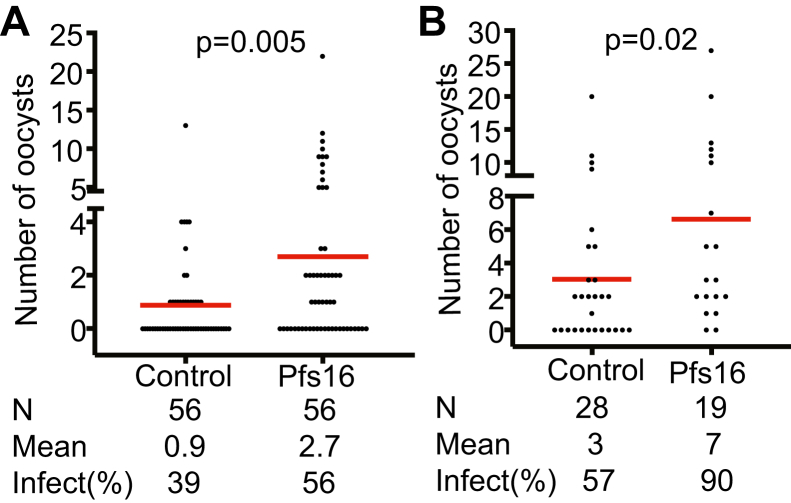
**Feeding mosquitoes with rPfs16 significantly increased the number of oocysts in *Anopheles gambiae* midguts.** (*A*) and (*B*) are replicates. About 1 × 10^5^ rPfs16-expressing Hi5 cells were suspended in 300 μl *Plasmodium falciparum*–infected blood containing about 1 × 10^5^ stage V gametocytes and fed *A. gambiae* to perform standard membrane-feeding assays. The same amount of Hi5 cells with empty vectors were used as the control. The Mann–Whitney U test was performed to calculate the significance of the oocyst numbers between the experimental and control groups. *n* = the number of midguts analyzed. The *red line* shows the mean oocysts. Mean: the average number of oocysts. Infect (%): percentage of infected mosquitoes. Hi5, High Five; rPfs16, recombinant Pfs16.

We examined the effect of Pfs16 on *P. falciparum* infection in mosquito midguts by feeding rPfs16. To keep the rPfs16 protein in its native membrane forms and mimic PVM whorls, we mixed about 1 × 10^5^ rPfs16-expressing Hi5 cells with 300 μl *P. falciparum*–infected blood containing about 1 × 10^5^ stage V gametocytes and performed standard membrane-feeding assays (SMFAs). The same number of Hi5 cells containing empty vectors was used as the control. The results showed that the number of oocysts per midgut significantly increased when compared to the control group. We observed the same results in two replicates, with one having higher infection prevalence than the other (Fig. 3). In addition, we examined the difference in infection prevalence and found that in the low and high infection experiments, oocyst prevalence significantly increased from 39% to 60% and from 57% to 90%, respectively (*p* < 0.05).

**Figure 4:**
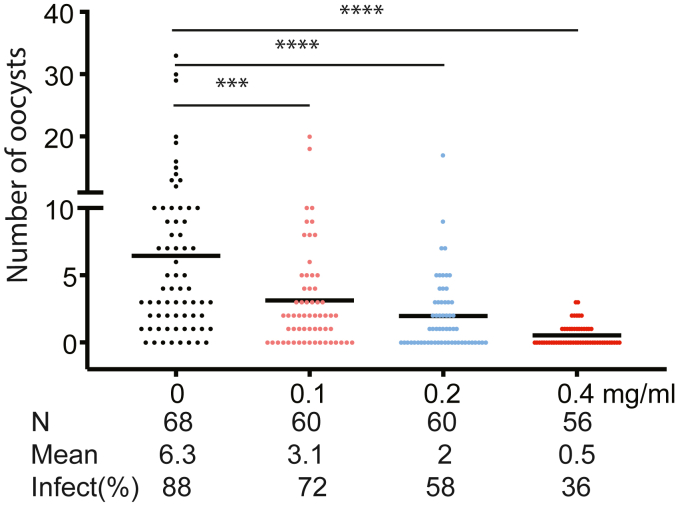
**Anti-Pfs16 polyclonal antibodies significantly reduced the number of*Plasmodium falciparum* oocysts in *Anopheles gambiae* midguts at a concentration-dependent manner.** Standard membrane feeding was performed with anti-Pfs16 antibodies at 0 mg/ml, 0.1 mg/ml, 0.2 mg/ml, and 0.4 mg/ml final concentrations. A commercial rabbit nonspecific IgG was used as the control to keep the final antibody concentration at 0.4 mg/ml. The Mann–Whitney U test was performed to calculate the significance between the oocyst numbers of experimental groups and the control. The chi-square test was used to calculate the difference in infection prevalence. ∗∗∗*p* < 0.001, ∗∗∗∗*p* < 0.0001. *N* = the number of midguts analyzed. The *red line* shows the median oocysts. Infect (%): percentage of infected mosquitoes.

Since our previous report identified that antibodies against Pfs16 inhibited *P. falciparum* transmission to mosquitoes (9), our initial hypothesis is that Pfs16 plays a similar role as FREP1, which anchors (32) and orientates parasites (33). If the initial hypothesis is true, the results from rPfs16 feeding assays were unexpected. Thus, we reexamined the effects of Pfs16-specific antibody-blocking assays. The purified rabbit polyclonal antibodies against Pfs16 were mixed with *P. falciparum*–infected blood and fed to mosquitoes to analyze antibody effects on malaria transmission. The purified rabbit nonspecific polyclonal antibodies were added to keep the total protein concentration at 0.4 mg/ml. Results showed that anti-Pfs16 antibodies significantly decreased the number of oocysts and infection prevalence in SMFAs (Fig. 4). We observed a dose-dependent decrease in oocyst load per midgut analyzed. The highest concentration had the highest inhibition. All concentrations of 0.1 mg/ml, 0.2 mg/ml, and 0.4 mg/ml were able to reduce oocyst load significantly (*p* < 0.001). The infection prevalence was calculated to be 88%, 72%, 58%, and 36% for the control, 0.1 mg/ml, 0.2 mg/ml, and 0.4 mg/ml groups, respectively. The prevalence decreased significantly compared to the control (∗*p* < 0.05, ∗∗∗*p* < 0.001, ∗∗∗∗*p* < 0.0001), calculated by the chi-square test. These results confirmed the previous report that antibodies against Pfs16 blocked malaria transmission (9). Therefore, Pfs16 must function beyond simple anchoring (33).

### Pfs16 exhibits adverse immunoregulatory properties

**Figure 5:**
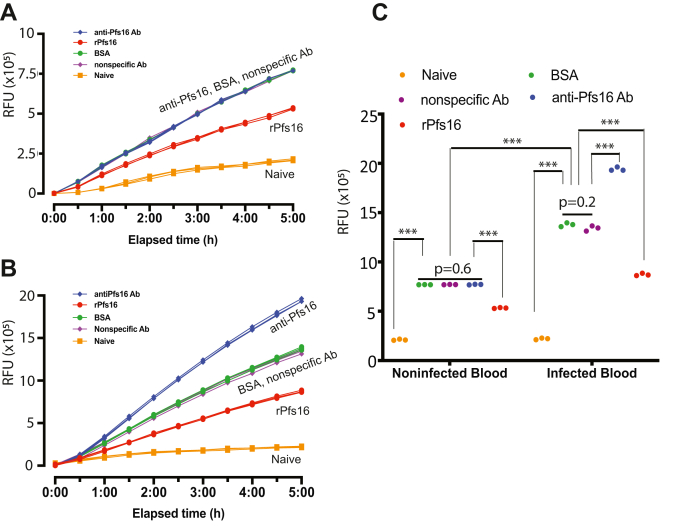
**rPfs16 protein inhibited *Anopheles gambiae* caspase activity in midguts.***A*, uninfected blood mixed with rPfs16, anti-Pfs16 Ab, BSA, and nonspecific Ab was used to feed mosquitoes. A significant decrease in caspase 3/7 activity was observed in the Pfs16 group only. The anti-Pfs16 Ab did not affect caspase 3/7 activities, same as the control, for example, BSA and nonspecific Ab. Blood-fed increased caspase activities compared with naïve mosquitoes. *B*, *Plasmodium falciparum*–infected blood mixed with rPfs16, anti-Pfs16 Ab, BSA, and nonspecific Ab was used to feed mosquitoes. A significant decrease in caspase 3/7 activity was observed in the Pfs16-treated group. Anti-Pfs16 antibodies removed the inhibition of endogenous Pfs16 in the infected blood on caspase 3/7 activity, showing significantly higher caspase activities. Infected blood-feeding increased caspase activities, compared with noninfected blood-feeding or naïve. *C*, the caspase activities in mosquito midguts 18 h after treatments. The development time of the caspase activity assay was 5 h. ∗∗∗*p* < 0.001. *n* = 5 midguts per group. BSA, bovine serum albumin; rPfs16, recombinant Pfs16.

Since anti-Pfs16 antibodies and rPfs16 protein showed opposite effects on *P. falciparum* transmission to mosquitoes, we hypothesized that Pfs16 might inhibit mosquito innate immunity. Therefore, we examined the effects of anti-Pfs16 antibodies and rPfs16 protein on the activities of caspase 3/7, key enzymes in JNK pathway, in *Anopheles gambiae* midguts of uninfected blood-fed, *P. falciparum*–infected blood-fed, and naïve mosquitoes. Bovine serum albumin (BSA) in PBS was used as a control. We used the uninfected blood-fed mosquito midguts as a baseline to investigate the caspase 3/7 activity. A nonrelated protein, BSA in PBS, and nonspecific rabbit IgG were used as a protein and antibody control, respectively. Mosquito midguts were collected 18 h after treatments, and the activities of caspase 3/7 in midguts were measured using Apo-ONE. The relative fluorescence units were measured every 30 min for 5 h to optimize the incubation time. For the uninfected blood-fed mosquitoes, blood-feeding increased caspase activities compared with naïve mosquito midguts. The caspase 3/7 activities in rPfs16-fed mosquito midguts were lower than those in the controls, for example, BSA and nonspecific antibody (Ab)-fed mosquitoes (Fig. 5*A*). There was no significant difference for the caspase 3/7 activities among anti-Pfs16 Ab-fed mosquito midguts, BSA, and nonspecific Ab controls, consistent to the fact that there was no Pfs16 in uninfected blood.

In the *Plasmodium*-infected *A. gambiae* midguts (Fig. 5*B*), we observed that rPfs16 reduced caspase activities compared with those in BSA and nonspecific Ab-fed controls. Different from noninfected blood, the infected blood contained endogenous Pfs16. Results show that upon adding anti-Pfs16 Ab, which neutralized endogenous Pfs16, the caspase activity in anti-Pfs16 Ab-treated midguts was the highest of all (Fig. 5*B*).

Statistical analyses of all the treated mosquito midguts showed that rPfs16 significantly reduced caspase activities (*p* < 0.0001) compared with the controls, for example, BSA and nonspecific Ab-fed mosquitoes. Anti-Pfs16 significantly increased caspase activities for the infected blood-fed mosquitoes but not the uninfected blood-fed mosquitoes. Analyzing the infected blood-fed and the uninfected blood-fed *A. gambiae* midguts, by comparing Figure 5, *A* and *B*, also revealed that *P. falciparum*–infected blood significantly increased caspase activities in mosquito midguts, compared with noninfected blood, which is consistent with the fact that *P. falciparum* invasion stimulates mosquito immunity. Collectively, *P. falciparum* Pfs16 inhibits the activity of caspases, components of mosquito immune response.

### Insect cell–expressed rPfs16 interacts with the *A. gambiae* midgut

**Figure 6:**
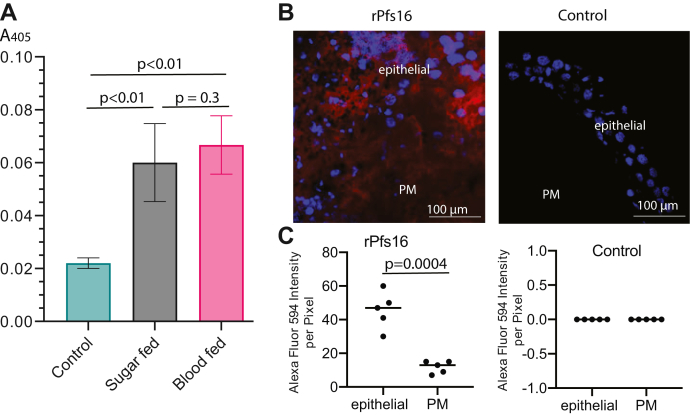
**Pfs16 interacts with mosquito midgut epithelial cells.***A*, ELISA showed that rPfs16 binding to either sugar-fed or blood-fed mosquito midguts was significantly higher than the BSA-coated control (*p* < 0.01). No significant difference between sugar-fed and blood-fed mosquito midguts. *B*, confocal immunofluorescence microscopy showed rPfs16 (*red*) binding to the blood-fed mosquito midgut epithelial cells, not the peritrophic matrix. Substitution of rPfs16 with BSA was used as the control. Only DAPI-stained dsDNA in cells displayed (*blue*) under the microscope. *C*, the bound rPfs16 to the epithelium is significantly more than that to the peritrophic matrix. The average pixel intensity was calculated using photoshop. BSA, bovine serum albumin; DAPI, 4′,6-diamidino-2-phenylindole; PM, peritrophic matrix; rPfs16, recombinant Pfs16.

Since Pfs16 inhibits caspase activities of midgut cells, it should interact with epithelial cells. This is different from the previous report that suggested Pfs16 interacts with peritrophic matrix (9). We reexamined this interaction with ELISA. We fed 3- to 5-day-old mosquitoes with blood and removed the blood bolus from mosquito midguts 24 h post blood meal. The isolated naïve and blood-fed mosquito midguts were homogenized, and supernatants (1 mg/ml proteins) were used to coat ELISA plates, followed by incubation with rPfs16. The retained rPfs16 was detected by rabbit polyclonal anti-Pfs16 antibody. The wells coated with BSA were used as the control. ELISA assays showed that the bound rPfs16 in both naïve and blood-fed midgut lysate-coated wells was significantly higher than the control (Fig. 6*A*). When comparing blood-fed *versus* sugar-fed midgut lysates, there was no significant difference. Since sugar-fed mosquitoes do not have a peritrophic matrix while blood-fed mosquitoes do, this result suggests that rPfs16 binds to molecules from epithelial.

Blood-fed mosquito midgut sections were incubated with rPfs16 and detected with anti-Pfs16 antibody using indirect immunofluorescence assay. Substitution of rPfs16 with BSA was the negative control. Results showed that the rPfs16 protein (red color) bound to broad areas inside the *A. gambiae* midgut when compared to the BSA control (Fig. 6*B*). Specifically, the fluorescence intensity of epithelial cells is significantly stronger than that in the peritrophic matrix (Fig. 6*C*). This result is consistent with the ELISA result and our previous actual data (9), i.e., Pfs16 bound to molecules at the epithelial cell layer, although we cannot exclude interactive molecules, which may also exist in the peritrophic matrix.

## Discussion

Malaria remains a leading disease. Besides regular vaccines to prevent parasites from infecting humans, many studies over the last decade also focused on vaccine development to block malaria transmission, for example, transmission-blocking vaccines, and discovered some key targets such as Pfs25, Pfs47, and α-tubulin-1 from the parasite (12, 33, 34, 35) and AnAPN1 and FREP1 from the mosquito (32, 35, 36, 37, 38, 39). Blocking malaria transmission by small molecules is also under investigation, and several fungal secondary metabolites have been identified (40, 41, 42).

The molecular mechanisms of parasite invasion inside mosquito midguts remain elusive. Recently, Pfs16 was reported to interact with mosquito midguts (9). Pfs16 is localized to the PVM with two regions across the PVM, consistent with a previous report (43). After egress, most Pfs16 protein found in the membrane remains termed multilaminated whorls (14), which ruptures into multilaminar vesicles (44, 45). Since protozoan parasites are known to be resourceful to be effective invaders/evaders, it is plausible that the sexual stage parasites use the remaining multilaminated whorls as a type of extracellular vesicles or exosomes for communication, pathogenesis, and/or immune regulation (46, 47). Indeed, our data demonstrate that parasites use Pfs16 in PVM to modulate mosquito immunity.

In this work, we showed that Pfs16 was in the membrane, interacted with mosquito epithelial cells, reduced the caspase 3/7 activities, and increased oocyst load. Since caspases in the JNK pathway (22) are critical components of mosquito innate immunity, inhibition of caspase activity by Pfs16 will silence mosquito innate immunity and facilitate parasites for invasion. Consistent with the previous report (48), our data show that parasite invasion stimulated the mosquito's immune response. Therefore, blocking endogenous Pfs16 with polyclonal antibodies in *P. falciparum*–infected blood-fed mosquitoes increased the caspase 3/7 activities and decreased the number of oocysts in mosquito midguts, as observed in our correlating infection assay. Our results demonstrate that Pfs16 interacts with the midgut and has immunomodulatory properties.

**Figure 7:**
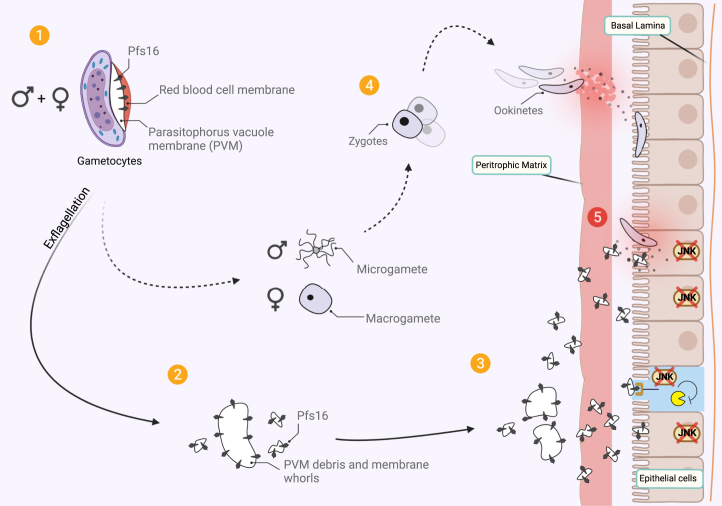
**Summary of suppression activity of Pfs16 in PVM remains on mosquito innate immunity.***1*, inside the infected mosquito, male and female gametocytes undergo exflagellation. *2*, the exflagellation process leaves the membrane remains with Pfs16 inside the membrane. The membrane reforms and breaks into smaller pieces to form PVM debris. *3*, the membrane whorls and PVM debris with Pfs16 travel through the peritrophic matrix and bind to the epithelial cells. *4*, in the meantime, normal parasite development occurs. A microgamete and a macrogamete form a zygote and transform into a motile ookinete that invades the midgut and challenges mosquito immunity. *5*, the Pfs16 from the gametocyte PVM remains starts the groundwork to inhibit the JNK pathway to silence innate mosquito immunity, which subsequently promotes ookinete survival and enables the parasite infection of mosquito midguts. JNK, Jun-N-terminal kinase; PVM, parasitophorous vacuole membrane.

In summary, we have demonstrated that *Plasmodium* parasites have evolved to utilize the Pfs16 in the remains of the PVM to silence the mosquito immune system and promote malaria transmission (Fig. 7). Therefore, Pfs16 is a promising target for transmission-blocking vaccines and drugs to control malaria.

## Experimental procedures

### Pfs16 sequence feature analysis

To determine the protein sequence features of Pfs16, we downloaded the protein amino acid sequence from PlasmoDB (https://plasmodb.org/plasmo/app/record/gene/PF3D7_0406200). The open-access AlphaFold2 Colab platform was used to predict the Pfs16 structure. The platform is available at https://colab.research.google.com/github/sokrypton/ColabFold/blob/main/AlphaFold2.ipynb. The Pfs16 amino acid sequence was used as an input, and automatic homology alignment was done with MMseqs2 and/or HHsearch embedded in the software coding. Signal peptide and transmembrane domains were predicted by SignalP (28) and TMHMM (29).

### Rearing mosquitoes

The *A. gambiae* (G3 strain) was obtained from the Biodefense and Emerging Infections Research Resources Repository and maintained in a temperature-controlled insectary that is set to 27 °C and 80% relative humidity on a 12 h day/night cycle. Larvae were fed with ground fish food, and adult mosquitoes were fed with a 10% sucrose solution. The commercially obtained human blood (Oklahoma Blood Institute) was washed with an equal volume of RPMI-1640 three times and collected by centrifugation at 500*g* for 5 min. The AB+ human serum (Interstate Blood Bank) was first heat-inactivated and mixed at a 1:1 ratio (v/v) with the previously washed blood. This mixture was used to feed mosquitoes to lay eggs *via* a glass feeding device (Chemglass).

### Culturing *P. falciparum*

The *P. falciparum* (NF54 strain) was obtained from the Biodefense and Emerging Infections Research Resources Repository. The parasites were cultured with RPMI-1640 (Gibco) complete medium containing fresh human red blood cells (4% O+, 10% AB+ serum, and 12.5 μg/ml hypoxanthine) in a candle jar at 37 °C. The culture was initiated at 0.25 to 0.5% parasitemia, and the medium was replaced daily. To collect parasites for SMFA, we centrifuged the 15- to 17-day-old parasites at 500*g* for 3 min and resuspended cells in human AB+ serum and O+ red blood cells mixed as described above.

### Generation of the rPfs16 protein

Total RNA was extracted from a *P. falciparum* (NF54) culture using RNAzol and its established protocols (Sigma-Aldrich). Complementary DNA was synthesized using the SuperScript First-Strand Synthesis System and established protocols (Invitrogen). The Pfs16 DNA product was amplified from the complementary DNA by PCR using the Phusion DNA Polymerase kit (Thermo Fisher Scientific) and gene-specific primers. The Pfs16 DNA fragment was purified by the GeneJet PCR Purification Kit (Thermo Fisher Scientific) and cloned into the modified plasmid pFastBac1 vector. We engineered the vector to include a 6xHis tag at the C terminus of the multiple cloning site. The final recombinant bacmid construct was created as described in the literature (https://www.thermofisher.com/document-connect/document-connect.html?url=https://assets.thermofisher.com/TFS-Assets%2FLSG%2Fmanuals%2FMAN0000414_BactoBacExpressionSystem_UG.pdf). In short, Pfs16 PCR-positive recombinant pFastBac1 plasmids were confirmed by sequencing and then transformed into DH10Bac competent cells to obtain recombinant bacmids. The ultrapure recombinant baculovirus construct DNA was extracted from DH10Bac *E. coli* with the PureLink HiPure Extraction kit (Invitrogen). PCR and sequence-validated rPfs16 viral particles were generated using established protocols in insect cells (https://www.thermofisher.com/us/en/home/references/protocols/cell-culture/transfection-protocol/cellfectin-2-reagent.html). High-titer recombinant viruses were generated in *Spodoptera frugiperda 9* cells and used to infect BTI-Tn-5B1-4, commonly known as Hi5 cells, to express rPfs16 at a multiplicity of infection of 2.5. The cells were harvested 80 h after recombinant viral infection, and cells were harvested by centrifugation at 500*g* for 5 min at 4 °C. The cells were resuspended in native cell lysis buffer supplemented with protease inhibitor (Invitrogen) and sonicated (pause for 30 s every 10 s) for 5 min on ice. Cell debris was removed by centrifugation at 10,000*g* for 5 min, and the final recombinant protein concentrations in the culture medium and cell lysate were measured with anti-His mAb’s in a standard curve. The lysate was further used and applied onto a HisTrap 5 ml column at 2.5 ml/min flow rate. The protein was then purified with Äkta Pure FPLC and checked for purity *via* SDS-PAGE (Cytiva). The protein was stored at −80 °C or directly used.

### Detecting rPfs16 expression by ELISA

rPfs16 was expressed on a small scale, and the medium and lysate were separated as described above. Samples were taken at 72 h and 96 h postinfection with the recombinant baculovirus. A flat-bottom 96-well plate (Brand) was used to coat equal concentrations of 3 mg/ml protein from either lysate or medium in triplicates at room temperature (RT) for 1 h. The wells were blocked with 2% BSA in 1× PBS and 0.1% Tween-20 (PBST) for 1.5 h at RT. The wells were then incubated with anti-His mAb at 1:1000 dilution in 2% BSA for 1 h at RT. Following three washes with 1× PBST, the wells were incubated with alkaline phosphatase–conjugated anti-mouse secondary antibody at 1:10,000 dilution in 2% BSA for 30 min at RT. After washing with 1× PBST three times, ready-made pNPP substrate (Thermo Fisher Scientific) was added and incubated for 1 h at 37 °C protected from light. Post incubation, the plate was directly read at *A*_405_ with the Synergy H1 microplate reader (BioTek).

### Parasite lysis and extraction

The parasite culture (either ookinete induced or not) was collected by centrifugation at 2000*g* for 5 min. The cell pellet was washed with 1× PBS three times and stored at −20 °C until use. Ookinetes were separated *via* the Percoll gradient and then extracted. To isolate the soluble protein fraction, the cell pellet was resuspended in a hypotonic buffer TEPI (10 mM Tris [pH 8.0], 1 mM EDTA, EDTA-free protease inhibitors) and repeatedly frozen and thawed, in total three times, before centrifugation at 16,000*g* for 10 min at 4 °C. After the soluble fraction was collected, the pellet was washed two times with TEPI, and the insoluble fraction was lastly extracted in NETT buffer (150 mM NaCl, 5 mM EDTA, 0.5% Triton X-100, and 50 mM Tris [pH 8.0]).

### Pfs16 immunoblots

The purified rPfs16 protein or the lysed parasite fractions were analyzed on a self-cast 15% SDS-PAGE gel with the Mini-Protean Tetra system according to the manufacturer protocol (Bio-Rad). The proteins were transferred onto a nitrocellulose membrane *via* the Mini Trans-Blot system for 1.5 h at 4 °C. The membrane was blocked for 1 h with 5% blocking milk in 1× PBST and then incubated with anti-Pfs16 at 1:300 in 5% blocking milk overnight at 4 °C. After three washes with 1× PBST, the membrane was incubated for 1 h at RT with alkaline phosphatase–conjugated secondary rabbit antibody at 1:30,000 dilution (Sigma). The blot was then washed three times with 1× PBST and soaked in an alkaline phosphatase solution for 10 min. The nitroblue tetrazolium/5-bromo-4-chloro-3-indolyl phosphate solution was added to develop the blot, and the image was taken using the Bio-Rad GelDoc Go.

### Mosquito lysis preparation

Fifty mosquito midguts were dissected 18 to 20 h post blood-feeding. The blood bolus was carefully removed while keeping the cleaned-out midguts in 1× PBS supplemented with protease inhibitor. Fifty sugar-fed mosquito midguts were dissected after 2 to 3 days post sugar feeding and kept in 1× PBS. After removing residual 1× PBS, the midguts were resuspended in 300 μl NETT lysis buffer and homogenized using a mini pestle three times for 10 s each and 30 s stop. Midgut tissues were further ultrasonicated for 90 s (10 s on and 30 s off) and centrifuged at 14,000*g* for 10 min. The supernatant was collected, and concentration was checked with a Synergy H1 microplate reader (BioTek).

### ELISA interaction assay

Flat-bottom 96-well plates (Brand) were coated in triplicate with 50 μl/well of blood-fed and sugar-fed midgut homogenates at 1 mg/ml proteins in solubilization buffer (15 mM Tris pH 8, 150 mM NaCl, and 5 mM EDTA with Pierce Protease Inhibitors) overnight at 4 °C. Three wells coated with 50 μl/well 1 mg/ml BSA in solubilization buffer were used as the control. The plate was then washed with 0.2% PBST (1× PBS and 0.2% Tween-20) and blocked with 2% BSA in 0.2% PBST for 1.5 h at RT. Then the ELISA plate was incubated with rPfs16 (0.5 μg) for 16 h at 4 °C. After three washes with 0.2% PBST, the plate was incubated with 1 μg/ml mouse monoclonal anti-6xHis antibody for 1 h at RT. Another three washing steps with 0.2% PBST were performed, and the plate was incubated with 0.2 μg/ml goat anti-mouse antibody conjugated to alkaline phosphatase. After the last three washing steps, the detection was performed with a p-nitrophenyl disodium phosphate-ready solution (Sigma). After 1 h incubation at 37 °C and protection from light, the absorbance at 405 nm was read with the Synergy H1 microplate reader (BioTek).

### rPfs16 immunofluorescence-binding assay to *A. gambiae* midguts

Twenty-seven hours post blood-feeding, *A. gambiae* midguts were isolated from 3- to 5-day-old mosquitoes. The blood bolus was carefully removed while keeping the cleaned-out midguts in 1× PBS supplemented with protease inhibitor. Either rPfs16 or BSA was added to the midguts at 10 μg/ml final concentration and incubated for 1 h at RT. After washing the midguts with 1× PBST three times for 5 min, the anti-Pfs16 pAb was added at 1 μg/ml for 1 h at RT with delicate shaking. After more washing, the Alexa Fluor 594–conjugated anti-rabbit secondary antibodies were added at a 1:500 dilution in 2% BSA and incubated in the dark for 1 h at RT. The treated midguts were air-dried and mounted with ProLong Gold containing 4′,6-diamidino-2-phenylindole nuclear stain. The examination occurred with an Olympus Fluoview confocal fluorescence microscope. Images were taken using sequential acquisition with variable z-steps and processed using Fiji/ImageJ software version v1.53c (https://imagej.nih.gov/ij/download.html).

### Standard membrane-feeding and protein-feeding assays

The SMFA was carried out as described previously (49). In short, the polyclonal anti-Pfs16 antibody was generated in rabbits and purified with immunogen affinity (Boster Bio). A 15- to 17-day-old *P. falciparum* culture was used to infect the mosquitoes with purified anti-Pfs16 rabbit polyclonal antibody in 1× PBS mixed with infectious blood (final antibody concentrations are 0.1 mg/ml, 0.2 mg/ml, and 0.4 mg/ml, respectively). The purified rabbit IgG control polyclonal antibody (proteintech) was used as the control to keep total antibody concentration at 0.4 mg/ml. After feeding, only the engorged mosquitoes were kept and maintained on a 10% sucrose solution. Seven days postinfection, midguts were dissected, stained with 0.1% mercurochrome, and examined using a light microscope to count oocysts. In case of the protein feeding assay, mosquitoes were fed with purified rPfs16. The rPfs16 protein was generated and purified as described in this article.

### Caspase 3/7 assay

Mosquitoes were fed with either *Plasmodium*-infected blood or uninfected blood. Caspase 3/7 activity was measured with the Apo-ONE Kit 18 h postinfection according to the manufacturer's protocol (Promega). Mosquitoes were dissected in groups of five midguts for every sample in ice-cold 1× PBS. The blood bolus was carefully removed. Midguts were collected in supplied lysis buffer to preserve caspase activity and kept on ice at all times. Midguts were homogenized *via* vortex and pestle and centrifuged at 10,000*g* for 5 min. The supernatant was added to a black 384-well plate in triplicates per group, and the caspase substrate Z-DEVD-R110 master mix was added at a 1:1 ratio. The plate was agitated at 350 RPM for 1 min to mix the contents. Fluorescent readings were taken at 499/521 nm for 5 h every 30 min with the Synergy H1 microplate reader set to 25 °C (BioTek). Blank was subtracted from the data points to get the net fluorescence value.

### Statistical analysis

All statistical analysis was carried out using GraphPad Prism, Version 9.4.1. The significance for the membrane-feeding assays was calculated with the nonparametric Mann–Whitney U test. Infection prevalence significance was calculated using the chi-square test. The caspase assay significance was calculated against vehicle control using a multiple paired *t* test among the individual time points.

## Data availability

All data generated or analyzed during this study are in this published article.

## Conflict of interest

The authors declare that they have no conflicts of interest with the contents of this article.
